# Integrating Smart Home Technology with Social Services: A Qualitative Study of Chinese Older Adults’ Experiences with the Care-on-Call Services

**DOI:** 10.3390/healthcare14101311

**Published:** 2026-05-12

**Authors:** Jianling Liang, Jie Zhuang, Jia Zhuang, Hok Bun Ku

**Affiliations:** 1 Department of Social Work, Wuyi University, Jiangmen 529020, China; liangjianling.liang@connect.polyu.hk; 2 Peking University – The Hong Kong Polytechnic University China Social Work Research Center, Beijing 100871, China; hok.bun.ku@polyu.edu.hk; 3 Department of Applied Social Sciences, Faculty of Health and Social Sciences, Hong Kong Polytechnic University, Hong Kong 999077, China; alexjia.zhuang@connect.polyu.hk

**Keywords:** smart home technology, social service, older adult users, Chinese eldercare, qualitative study

## Abstract

Background: Although the application of smart home technology in the Chinese eldercare market is widespread, its effectiveness from the users’ perspective remains underexplored. This qualitative study examines the perceptions and experiences of older adult users in adopting and applying the Care-on-Call services (Ping An Tong; PAT), a prominent example of smart home technology for eldercare in Mainland China. Methods: Individual and dyadic interviews were conducted with 28 older adult users from diverse physical, socioeconomic, and familial backgrounds. Thematic analysis was performed. Results: Two overarching themes were illustrated based on thematic analysis. First, the multifaceted challenges of using PAT encompass an incomplete cognition of the services, unfamiliarity with PAT systems, psycho-cultural resistance, ‘do it yourself, don’t bother others’, economic concerns of additional costs, and ethical concerns regarding information security and privacy. Second, bridging the technology divide highlights the empowerment of PAT use among older adults through a variety of educational methods to effectively utilize the services, enhancing service effectiveness through the integration of smart home technology and social service provision, and increasing service accessibility through inclusive services. The disparities in smart home technology application between China and the West are also discussed. Conclusions: Psychosocial support, organizational programs, and the integrated service model are recommended to promote the utilization of smart home technology among older adults in China.

## 1. Introduction

The new millennium has witnessed dramatic demographic transitions across the globe. When the number of older adults surged and the products and services the market offered to this group advanced, the focus of scholarly discourse on eldercare shifted. More recent discussions have focused on the application of various technologies in eldercare [1,2]. Among them, smart home technology (or smart homes), defined as a residence equipped with computing and information technology to anticipate and respond to the needs of the residents and promote social, health, emotional, economic, sustainability, and security benefits through the management, monitoring, support, and responsive functions within the home and connections to the external community, has gained significant attention [3,4].

Empirical studies, primarily from the perspectives of service developers and providers, most in Western contexts, have explored the benefits and barriers of smart home technology in eldercare [5,6]. On the positive side, smart home technology offers various biopsychosocial and financial benefits, particularly for older adults with health concerns. It enhances the biopsychosocial well-being of its users through multifarious supportive services and provides key health-related advantages, such as better care accessibility, improved safety, remote monitoring, social connectivity, and emotional support [7]. It is revolutionizing eldercare by empowering older adults’ sense of autonomy, independence, and connectivity [8].

Despite the potential benefits of smart homes, several barriers have been identified [9]. Technological barriers, including perceptions of compatibility, connectivity, and system reliability, are believed to significantly influence users’ views on the technology’s usefulness [10]. Usability—particularly reliability and ease of use—might also shape potential users’ acceptance of the technology [11]. The low perceived usefulness of smart homes often results from a lack of knowledge, trust, and experience [12]. Financial concerns, including the costs of installation and maintenance, might further deter adoption [13]. In addition, ethical concerns arising from the extensive collection and storage of personal data may pose privacy, security, and legal challenges, particularly in healthcare contexts [14]. Lastly, users’ fears of isolation and reduced human interaction might further hinder their acceptance [15].

Within the fields of information systems and technology, as well as organizational behavior and management, two commonly used theories to comprehend individuals’ application of technologies are the Technology Acceptance Model (TAM) [16] and the Unified Theory of Acceptance and Use of Technology (UTAUT) [17]. Both TAM and UTAUT suggest that individuals’ behavioral intention to use a technology defines their actual application of the technology. For TAM, intended use is determined by perceived usefulness and ease of use. Built upon TAM, UTAUT proposes four constructs that affect usage intention—performance expectancy, effort expectancy, facilitating conditions, and social influence. The first three factors concern one’s subjective evaluation of the technology and its supporting systems and infrastructure. Performance expectancy refers to individuals’ confidence in the technology system’s ability to help them achieve expected outcomes; effort expectancy refers to the belief in the ease of using the system; and facilitating conditions refer to individuals’ assessment of the organization’s technical infrastructure that supports the use of the technology. The social influence dimension concerns individuals’ perception of the external social environment, specifically whether significant others believe that they should use the technology. The theory posits that the four factors together predict individuals’ behavioral intention to use a technology, which in turn predicts their actual use behavior. It further highlights the importance of individual characteristics in moderating the associations between the four key predictors and willingness to use the technology [18].

While TAM and UTAUT are recognized for providing meaningful insights into how users interact with technology [19], they are often criticized for focusing too much on users’ assessments of technology and assuming a direct causal influence from intention to actual use behavior [20]. A shortcoming of the two models is their neglect of broader system-level factors that extend beyond the technology and users’ acceptance and satisfaction with it. A more comprehensive understanding of individuals’ application or intention to use the technology should also incorporate their consideration of factors from external social systems into their evaluation and use of the technology [21]. Thus, the complexity of the socio-technical system includes technological elements (e.g., system features and functionality, interoperability, and usability) as well as organizational and social components, such as social norms and appreciation of the technology, trust of the technologies, opinions from peers, suggestions from professionals, and so forth [22].

In this light, Schulz et al. asserted that research on technology adoption among older adults should go beyond describing facilitators and barriers and necessitate a comprehensive understanding of their social environment and interactions with it [23]. Schroeder et al. further identified social influence (e.g., subjective social norms, social capital, and interpersonal relationships) as a critical determinant of older adults’ intention to use technology [24]. Specifically, recent studies have highlighted that trust is a crucial factor in the adoption of technology among older adults as it directly influences acceptance and willingness to engage with new systems [25]. A lack of trust might lead to skepticism about the efficacy and security of technologies, ultimately hindering their successful implementation and use in healthcare settings [21]. Another often-discussed factor is the cultural values and beliefs of the community, which significantly influence individuals’ intention to use technology by shaping values, beliefs, and social norms that affect perceptions of usefulness and ease of use [26].

With the development of smart home technology, wearable sensors, remote monitoring systems, and digital health platforms have been pioneered to increase the health of service users [27]. The systems provide clinically oriented measurement of health-related signals and enable care delivery through integrated analytics, alerts, and follow-up actions, supporting healthcare decision-making rather than home automation alone, and aiming to increase trust in the technology.

In China, a range of supportive services has been provided by the state, the market, and non-governmental organizations to enhance the quality of life of older adults and address their unique biopsychosocial needs [28]. Ping An Tong (PAT) (Care-on-Call services) is a prominent example of smart home solutions for eldercare in Mainland China. The PAT service in Jiangmen City, Guangdong Province, is offered by a Social Work Integrated Service Center.

The service is built on the Internet of Things, information technology, big data, cloud computing, and other technologies in eldercare. The hardware components of the system platform mainly include the PBX all-in-one softswitch server (call server), the voice storage server, the network storage server, the disk array server, the telephone exchange, the switch, and the UPS uninterruptible power supply. The basic PAT service will install a landline phone at the user’s home. Using the red emergency assist button and the green regular service button on the phone, as well as the waterproof remote control at home, users can contact the PAT center for service. Launched in September 2015, the PAT service has since covered 63 towns and streets in Jiangmen City. It creates a unified platform operation across three districts and four cities in Jiangmen City. The service primarily targets older adults in need, including low-income families, older adults living alone, those living with a spouse, those who have lost their only child, and those with disabilities. The government provides funding to offer them free basic PAT services. It served over 28,000 people until 2024, with more than 3.06 million service instances recorded.

The PAT service provides integrated supportive services for older adults and people with disabilities. This includes 24/7 emergency and health services (e.g., appointment scheduling, post-hospital follow-ups, location tracking, and year-round weather disaster alerts), home-based daily life support (e.g., age-friendly renovations, housekeeping, property maintenance, procurement of daily necessities, gas supply, and medication purchasing), consultancy services (e.g., policy advice, eldercare consulting, and rights protection), and emotional support services (e.g., phone check-ins and emotional counseling). Its offline services include home visits, health education, recreational activities, and community volunteer visits, all as part of a one-stop integrated smart home technology service.

Despite the substantial research on smart home technology, four key knowledge gaps remain regarding the effectiveness and application of smart homes in eldercare. First, existing studies have obtained data from ‘smart home’ developers and providers, while the service users’ voices are always neglected [12,29]. Second, previous research has focused more on the intentions to use smart home technology, whereas a more comprehensive understanding of smart home applications in eldercare necessitates an in-depth investigation of users’ views and experiences after implementation [14,30]. From the perspective of practice research, the experiences and insights of service users not only uncover the specific needs and preferences of the target population but also guide changes in practice and policy, advocating for necessary adjustments informed by user feedback [13]. Third, most studies have been conducted in Europe and the United States. Cultural and regional differences also require more attention. Although more and more smart home technologies are being used in the Chinese eldercare market, there are few studies on effectiveness. Fourth, system-level issues, including organizational and social components and service users’ interactions with the social environment, require greater attention. In this light, the current study aims to address these knowledge gaps and contribute to the literature by exploring three research questions, focusing on the experiences and perspectives of older adults using smart home technology in the Chinese context:

RQ1: How do Care-on-Call older adult users perceive and experience the services in their daily care and living?

RQ2: What specific facilitators, barriers, and unmet needs emerge from the lived experiences of older adult users interacting with the Care-on-Call services?

RQ3: How do cultural, social, and familial factors shape older adults’ integration and application of the Care-on-Call services?

## 2. Methodology

### 2.1. Design

This study employed a qualitative approach using in-depth interviews to explore the experiences and perceptions of older adult users in using PAT services in Jiangmen City. A qualitative approach was adopted because it emphasizes the views of fewer people in greater depth and focuses on building a convincing analytical narrative [31]. It facilitates researchers to explore and gain a rich understanding by revealing the individuals’ authentic experiences.

### 2.2. Participants

Purposive sampling, particularly maximum variation sampling, was applied because it enables the research team to efficiently recruit older adult users covered in the PAT service with diverse health, socioeconomic, and familial backgrounds. Factors such as gender, age, familial structure (i.e., living alone, living with a spouse, and living with children), marital status, having adult children, health condition, occupation before retirement, and having pension and subsidies may have a potential impact on older adults’ experience and perceptions of using PAT services. They are considered in purposive sampling. The inclusion criteria for participants were (a) aged ≥60, (b) using PAT for over six months, (c) having stable psychiatric conditions, (d) able to communicate, and (e) willing to take part in the study. The exclusion criteria were older adults with cognitive impairment that resulted in an inability to communicate. The research team provided the sampling criteria to the center which provides PAT services. With the help of the center, the research team contacted potential participants and invited them to participate in the study. The researchers recruited participants covering all the factors listed in the sampling criteria to achieve full representation.

### 2.3. Data Collection

Interviews were conducted in September 2024. All interviews were performed in the participants’ homes. All interviews were audio-recorded, and each lasted 45–60 min. The semi-structured interviews were conducted guided by the integrated theoretical framework. In practice, participants were invited to reflect on their perceptions and experiences of using PAT services, including their intentions of adopting PAT, initiated service-seeking behavior, physical, psychological, social, and financial influences on service usage, impressive experiences of service use, the effectiveness of the services, interactions with service providers, social influences on applying PAT (i.e., broad cultural values and beliefs of the community or significant others), and their expectations of improvements in PAT services. The data collection process (i.e., interviews) was conducted until data saturation was reached, that is, when the interviews no longer elicited new data [32]. In total, 20 individual interviews and 4 dyadic interviews were conducted before saturation was tentatively reached, and the assessment continued until no new themes emerged. Participants were allowed to share at their own pace, enabling the researchers to gain a comprehensive understanding of their experiences and perceptions regarding the application of PAT services. Individual interviews were conducted with older adults living alone or with children, and dyadic interviews were conducted with those living with a spouse. The first, corresponding, and third authors participated in the interviews.

### 2.4. Research Ethics

The study was approved by the institutional review board of the corresponding author’s university. The purpose, content, and format of the study were comprehensibly explained to potential participants in their mother tongue, and confidentiality, voluntary participation, and protection of human rights were addressed. Written informed consent was obtained from the participants before data collection. All participants were assured of their rights and freedom to withdraw and to request the deletion of any data and research information from the study, without any obligation or penalty. All data collected was stored and treated with strict confidentiality. All data will be deleted five years after the project ends. Only the researchers of this study had access to the data. To further protect participants’ privacy and confidentiality, each participant was represented by a code in the data and interview transcripts.

### 2.5. Data Analysis

All interviews were audio-recorded with the participants’ consent and transcribed verbatim. Thematic analysis was applied to identify, analyze, and interpret data in this study, and seven procedures were followed [33]. The researchers read and reread all 24 transcripts (20 individual and four dyadic interviews) to become familiar with and pay special attention to the patterns that emerged. The initial codes were generated and combined into overarching themes. The researchers coherently recognized how the themes support the data and the theoretical perspective, comprehensively defined each theme, and created a thick description of the findings. For instance, a prevailing attitude of ‘do it yourself, don’t bother others’ and the view ‘beg for help’ towards the PAT services were identified as codes, which were systematically organized and grouped into a subtheme as ‘psycho-cultural resistance’. Subthemes of cognitive insufficiency, technological barriers, psycho-cultural resistance, and economic and ethical concerns were identified and combined to create the larger theme ‘multifaceted challenges of applying PAT’.

The analysis was iterative and ongoing. The first, corresponding, and third authors regularly discussed issues related to the data analysis process, including how the initial codes, subthemes, and themes were generated and the meaning of each code, subtheme, and theme, until agreement was reached. Disagreements were resolved through discussions with the fourth author.

### 2.6. Rigor

#### 2.6.1. Reflexivity

Researchers’ influence on data collection and analysis is unavoidable in qualitative research. Therefore, knowledge of the researchers’ backgrounds would be useful for readers to better understand data collection and interpretation in qualitative research.

The first and corresponding authors are registered social workers with considerable experience conducting qualitative research. The first author had nearly 20 years of experience in health and gerontological social work practice and had been a university social work lecturer for almost 10 years. The corresponding author has been a medical and gerontological social worker for over 10 years and is now a university researcher specializing in qualitative research in health and gerontological social work. The first and second authors brought an insider perspective, fostering deep rapport and sensitivity to the unique needs of the older adult participants. Their professional identity also facilitated communication and trust during data collection. However, we remained critically aware that this insider status could potentially influence assumptions and interpretations. To mitigate this, the data collection and analysis were structured under the guidance of the fourth author, a senior university professor specializing in qualitative and practice research, who provided training and facilitated rigorous and collaborative analysis. Furthermore, the inclusion of the third author, with a background in mixed-methods and social policy research, encourages the team to consider broader social contexts when interpreting the data. Throughout the study, the research team engaged in reflexive discussions to examine how our collective experiences in social work practice, teaching, and qualitative inquiry informed every stage, from interview questioning to thematic analysis, ensuring that the findings remained grounded in participants’ narratives rather than in our pre-existing professional frameworks.

#### 2.6.2. Trustworthiness

Multiple strategies, guided by Lincoln et al., were adopted to ensure the trustworthiness of this research, including credibility, dependability, confirmability, transferability, and authenticity [34]. Credibility was achieved through triangulation of our interview data with the research team’s on-site observations and notes, as well as peer debriefing sessions. We have also discussed our preliminary results with a couple of frontline practitioners in the field to challenge our interpretations. Confirmability was obtained by audit logs and inter-coder agreement. Dependability was fulfilled through the researchers’ reflexivity to mitigate research bias and by maintaining documentation for how interpretations were formed. Transferability was achieved through our maximum variation sampling to capture diverse experiences and through thick, contextual descriptions of the participants and setting. The provision of a rich, detailed description is emphasized to address authenticity.

## 3. Results

### 3.1. Characteristics of the Sample

In total, 28 older adults from 24 households were interviewed, including 11 living alone, 8 living with their families, and 9 living with a spouse. Table 1 details the demographic features of the 28 participants. The study consisted of fourteen females and fourteen males. Most participants were in their 80s. Twelve participants were married, eleven participants were widowed, three participants were divorced, and two participants were single. Nineteen participants had chronic conditions. In total, 39.3% of participants had pensions, ranging from 3000 to 4000 CNY. Seven participants (25%) received poverty, government subsidies, and disability allowances.

### 3.2. The Experience and Perceptions of Participants

This study illustrates a comprehensive understanding of Chinese older adults’ experiences and perceptions of using PAT. Two overarching themes emerged from the thematic analysis: (a) ‘multifaceted challenges of applying PAT’ and (b) ‘bridging the technology divide’. The main themes and corresponding subthemes are shown in Table 2.

### 3.3. Multifaceted Challenges of Applying PAT

This study identified five subthemes of challenges that impede post-implementation usage among PAT users: cognitive insufficiency, technological barriers, psycho-cultural resistance, and economic and ethical concerns.

#### 3.3.1. Cognitive Insufficiency

The first subtheme is users’ cognitive insufficiency. They had a limited understanding of comprehensive services. The PAT services in Jiangmen City have evolved significantly from their initial focus on emergency assistance to a comprehensive, one-stop, integrated social service accessible both online and offline. However, many participants remain unaware of the full scope of services available. While almost all participants recognized the emergency and gas supply services, only five had a relatively complete picture of PAT’s additional services. Over 40% of participants indicated that they believe that PAT should be used only in emergencies.

I do not really know what kind of services there are. I called them only when I really needed it.

(A10)

Nearly 20% of the participants expressed concerns about contacting PAT, fearing that they might cause unnecessary trouble for its staff, a significant issue for them. Beyond insufficient promotion of PAT, most participants were in their 80s and had relatively low literacy. Moreover, nearly two-thirds of the participants had chronic diseases, such as hearing, vision, and mobility dysfunction, which all affected their access and understanding of the services.

#### 3.3.2. Technological Barriers

The technological barriers—participants’ unfamiliarity with applying smart home technologies—also hinder their use of the services. The principal PAT technology at the user’s end comprises a home and portable device. The fundamental services are supported by the home device’s emergency and inquiry buttons, while the portable service includes only the emergency button. The volume on both devices is set to maximum to enhance communication between PAT staff and users. The simple yet thoughtful design of PAT aims to increase the feasibility and accessibility of its services. Nonetheless, when we invited participants to demonstrate how to operate their home devices, five participants failed. They admitted that although PAT staff introduced the methods of operation to them during home visits, they got lost easily.

It is just that I have a poor memory. Even if the lady (PAT staff) comes and says, ‘Auntie, press here, press there,’ I quickly forget, and sometimes I press the wrong button.

(A9)

#### 3.3.3. Psycho-Cultural Resistance

Furthermore, psycho-cultural resistance hinders participants’ application of PAT services. In our study, nine participants expressed a prevailing attitude of ‘do it yourself, don’t bother others.’ This mindset reflects a strong desire for independence and an avoidance of relying on others and of causing inconvenience to the staff. Among the nine participants, three had never initiated a call to PAT for help, although they were sometimes in real need. Six of them had used the PAT services more than once but showed a strong reluctance to do so.

I have always been the kind of person who doesn’t want to trouble others. If I can handle it myself, I won’t trouble others.

(A2)

One participant (A8) even used the word ‘beg for help’ to express her view on the PAT services. She claimed that she would not use the services unless necessary. If she failed to solve the problems, she would prefer to seek help from family members before ‘begging for help’ from PAT staff. Moreover, some participants were unfamiliar with PAT services and believed that it could only be used in life-threatening emergencies, which made them feel unlucky about using it.

#### 3.3.4. Economic Concerns

Despite the financial benefits PAT brings to its users in their daily lives by connecting them with third-party resources, financial concerns remain regarding the cost of the PAT service itself. In Jiangmen City, the eligibility criteria for free use of PAT’s basic services include age, economic status, physical condition, and family structure. Those who do not meet these requirements must cover the costs themselves. In the study, the participants are all eligible for the basic service. However, PAT has recently launched its innovative PAT watch and various other smart home devices, which require out-of-pocket expenses. When asked if they would use these new products, the vast majority of the participants declined, citing financial concerns. Most older adults opt only for the free basic PAT services. Only two participants reported using PAT watches, and one of them decided not to renew due to financial considerations.

Because I have to share one pension between two people, I will not renew the subscription for the watch functions that require additional fees beyond the basic PAT features.

(B2_M)

#### 3.3.5. Ethical Concerns

In narratives about PAT services, ethical issues emerge regarding the protection of privacy, confidentiality, and information security. While PAT connects to fixed-line phones, facilitating daily communication, it also produces ethical risks, such as phone fraud. Two of the participants reported receiving unknown and fraudulent calls. They expressed concern about the privacy and information security vulnerabilities associated with the PAT systems.

I’ve experienced receiving several calls from unknown numbers, often related to insurance sales and advertisements.

(C4)

### 3.4. Bridging the Technology Divide

Bridging the technology divide is another overarching theme from the users’ perspective regarding the adoption and application of PAT services. The study demonstrated that PAT focuses on bridging the technology divide through empowering the use of PAT, improving effectiveness through the integration of technology and social services, and enhancing accessibility through inclusive services.

#### 3.4.1. Empowering the Use of PAT

The participants were empowered through a variety of educational methods to effectively utilize the services, including regular phone check-ins, personalized home visits, and the thoughtful distribution of promotional materials. Just as B5_F said: ‘They call every month and even come in person’. Recently, the participants were motivated to connect with the PAT community by following the WeChat Official Account and adding a dedicated customer service WeChat account. This initiative facilitates convenient, timely communication, enabling them to easily reach out to PAT staff for support.

#### 3.4.2. Improving Effectiveness Through the Integration of Technology and Social Services

PAT services feature the integration of smart home technology and social service provision, emphasizing users’ physical, psychosocial, and financial needs, through diversified online and offline services supported by smart home technologies, as reported by participants.

*Basic physical benefits.* The core advantage of PAT lies in enhancing user safety. Emergency services are frequently highlighted by participants. PAT provides comprehensive 24/7 emergency support, ensuring prompt contact with ambulances and family members, and is complemented by timely follow-ups. Among the participants, four had utilized these emergency services, including three older adults living alone and one living with their spouse. Although over 80% of participants have not used emergency support services, they all have a clear understanding of when and how to use them.

When I feel unwell, I can just press the button (of PAT) to call for a ride to the hospital immediately, without having to search for nearby hospitals.

(A1)

Health monitoring and management are the second fundamental functions of smart home technology concerning users’ health [35]. It appeared to be an important PAT function favored by participants. In this study, nearly 70% of participants reported having chronic illnesses, such as hypertension, diabetes, and mobility dysfunction. They had concerns about accidental health crises. PAT checks its users’ physical condition, reminds them of daily living precautions, and records each older adult user’s health status on its information platform. Nearly one-third of the participants mentioned that the accessibility and availability of services maximize their sense of security in the community, especially for those living alone and living with a spouse. A10 was living alone and struggled with unstable blood pressure. He often felt anxious about being alone, fearing that ‘no one would know if something bad happened.’ However, this worry was alleviated after the installation of PAT. With the emergency support from PAT, A10 no longer feels the need to inconvenience his son whenever he experiences discomfort, a relief for both of them.

PAT also offers offline health services, such as regular home visits and community health workshops. Nearly 60% of our participants mentioned PAT’s offline services. B2_M articulated PAT’s health programs: ‘A family doctor would be there, and activities would take place in the activity room. Many times, when I saw the door open, I would just go to join it.’

*The feelings of being cared for.* In the study, nearly three-quarters of participants mentioned being proactively contacted by PAT for daily and holiday greetings, bad-weather reminders, and emotional support. They claimed that these warm gestures gave them a sense of being cared for, especially for those who live alone or with a spouse, to a certain extent, making up for their loneliness.

The best thing about PAT is its care for us, with messages like ‘Uncle, how’s your health? Is everything okay?’ That alone is enough for us.

(B3_M)

*Increasing social inclusion.* Our interviews revealed that 46% of participants who were relatively older and had physical limitations reported low engagement and high disconnection from their community. Yet PAT plays an alternative role in safeguarding their social inclusion. PAT employs a district management system, in which a team is assigned to make regular visits to its older adult users within a specific district. Over 20% of participants mentioned that home visits arranged by PAT staff were an important part of their social lives, and low staff turnover has enabled them to build trust. A11 lives alone and rarely goes out due to his vision impairment. Regular home visits by familiar PAT staff help him maintain a sense of belonging to the community. Acting as a medium, the service establishes a social network for him, enabling sustained social support and social inclusion.

When the PAT staff arrives, I can tell by the sound that she’s here. PAT seldom changes the staff, and they know me well, especially her, who visits me regularly.

(A11)

In addition to the sense of social connectedness fostered by PAT’s home visit services for older adults with limited health conditions, its community activities also enhance participants’ social well-being; around one-third reported participating in PAT-organized community activities. A3 lives alone on the sixth floor of a building without an elevator. She has never been married and has no children. She suffers from rheumatism, which limits her frequency of going out to only once a day for groceries, visiting family members, or taking short walks. Yet, the calls and home visits, along with the community activities organized by PAT, enrich her social life.

Sometimes, they call to inform me about their activities. They invite me to participate, and I do attend.

(A3)

*Daily life support.* Participants in the study underlined the financial benefit of PAT in their daily lives. Around three-quarters of participants noted that PAT has connected them with various community and social resources from third parties at lower cost, including housekeeping, property maintenance, procurement of daily necessities, gas supply, medication purchasing, and age-friendly home renovations and improvements, which reduce their economic burden, protect their rights and interests, and improve the quality of their everyday lives.

A striking example is its gas supply service, acknowledged by over half of our participants. PAT has partnered with the local gas company to provide a gas delivery service to its users at a price 15% lower than the market price. This service is particularly welcomed by older adult users who do not have pensions and rely on their savings and government subsidies. C4 was living together with his wife, son, and mother, and needed wheelchair assistance for daily activities. He had no pension but only a monthly disability allowance of 1000 yuan, and his wife worked as a sanitation worker and had no pension either. He thought the gas supply service was important: ‘it’s cheaper, so we can save a lot’.

Meanwhile, PAT provides services, such as home appliance repair, that prevents older adults from having to search for and compare relevant services on their own and further safeguards service users’ interests.

Sometimes, if the electrical appliances are not working properly, we call them again. I call PAT, and it will definitely be safe. If anything happens, PAT will hold me accountable.

(A9)

#### 3.4.3. Enhancing Accessibility Through Inclusive Services

One key point frequently emphasized by our participants was the inclusive features of PAT services, which they identified as a crucial motivation for their use. With the financial support from the local municipal government, PAT offers these services free of charge to older adults who meet specific criteria, and it has continuously adjusted its access criteria to benefit a larger number of older adults, including lowering the age requirement to 75 for those living alone or with a spouse. These inclusive services have significantly increased motivation to use PAT, particularly among vulnerable groups with limited financial resources. In our study, 25% of participants were receiving poverty and government subsidies or disability allowances. Nearly 40% lived alone, 32% lived with a spouse, and almost 80% were aged over 75. The inclusive features of PAT have enabled older adults, especially those with special needs, to gain fair access to essential services.

## 4. Discussion

Although smart home technology is widely used in the Chinese eldercare market, few studies have investigated its effectiveness and applications. Moreover, the experiences and insights of service users are also often neglected. Our study fills these knowledge gaps through a qualitative study of the application of PAT services in Jiangmen City from the perspectives of older adult users. Our interviews with participants from diverse family structures and socioeconomic backgrounds revealed a significant difference between China and the West in the application of smart home technology for older adults. This highlights the local practice of bridging the technological divide and enhancing the application of services among older adults in China.

### 4.1. Identifying Multifaceted Challenges of Applying Smart Home Technology

In examining the multifaceted challenges to the usage of the PAT service among older adults in Jiangmen City, it becomes evident that cognitive limitations significantly hinder effective utilization, while the cognitive challenges were considered not critical among older adults in Western contexts [12,36]. The low perceived usefulness of smart homes might be attributed to a lack of knowledge, trust, and experience in harnessing the technology’s benefits [3] and may stem from insufficient exposure to and promotion of smart home technology among Chinese older adult users. Technological challenges, including users’ incomplete understanding of the technology compatibility, reliability, automation, and interoperability, might obstruct their application of the technology [10]. Health deterioration in older adults, such as memory and mobility, may further limit their use of technology.

In the current literature, financial concerns about smart home technologies include the cost of the technology as well as the costs associated with its installation, repair, and maintenance [13]. In our research, although older adult users benefit from free installation and basic PAT services, they still find it difficult to afford advanced services that require out-of-pocket costs. Economic factors remain an unavoidable consideration in the adoption. Privacy concerns and information security have been identified as a barrier to potential users’ acceptance of smart home technologies [14,37], consistent with our findings. Especially for older adults who are not familiar with technology, this highlights their vulnerability to privacy and information security threats. Given the importance of data protection in smart healthcare systems, a more detailed consideration of cybersecurity measures and regulatory frameworks is recommended [38].

In addition, psycho-cultural attitudes significantly influence the willingness of older adults to seek assistance from the PAT service. The psycho-cultural challenges to smart home technology usage are described as individuals’ resistance to changing habits, behaviors, and lifestyles that are necessary to adopt the technology and utilize its services [39]. The prevalent mindset of self-reliance, characterized by a reluctance to ‘bother’ others, especially seeking help from social service organizations, reflects deep-rooted cultural values that prioritize independence and complying with the hierarchical structure among Chinese older adults [40]. Informal and official helping systems coexist in the Chinese mainland. The former, characterized by self-help and mutual assistance among family members, is shaped by a hierarchical structure rooted in family-centered values [41]. Before the 1980s reform of the social welfare system, the leading provider of social care in China was the family, with additional support from local authorities in rural regions and employers in urban areas [42]. From the beginning of the 21st century, there has been a structural shift in the promotion of social services for older adults, from an over-reliance on family to a ‘home-based, community service-reliant and institutional care-supplemented’ policy [28]. Government procurement of social services has fostered the development of NGOs delivering social services [43]. Guangdong Province is one of the pioneers in purchasing social services from non-governmental sectors [44]. Nevertheless, formal social service organizations are still underdeveloped in China, as many older adults tend to feel more comfortable and accustomed to relying on informal support networks [28]. Addressing these psycho-cultural barriers is essential for enhancing the acceptance and usage of PAT services.

### 4.2. Returning to the Essence of the Humanities to Bridge the Technology Divide

In terms of bridging the technology divide, this study demonstrates that even with the installation of smart home technology, users still require PAT staff to continually educate, encourage, and empower their usage, making the services more effective and beneficial for them. The key finding of PAT services reveals that smart home technology serves as a means, with service as its essence. The current literature highlights smart home technologies aimed at comfort, monitoring, and management for older adults, vulnerable individuals, and those with chronic conditions [30], whereas PAT focuses more on service-oriented features. PAT, as depicted by its users, is not only a technology platform that promotes independence, safety, and convenience, but also an integrated social service platform dedicated to addressing the multifaceted physical, psychological, social, and financial needs of older adults. The PAT service has developed an indigenous integrated model to address the challenges of using smart home technology.

In the current literature on smart homes, there has been a predominant focus on physical health and healthcare, while psychological well-being and social inclusion have received comparatively little attention [45,46]. Older adults’ interactions with the external environment are crucial to their functional capacity, which in turn significantly influences their biopsychosocial dimensions of well-being [47,48]. Empirical evidence indicates that older adults who engage in more frequent social activities report better self-rated physical health and lower rates of depression, dementia, and cognitive impairment [49,50]. PAT provides emotional support to promote psychosocial well-being through its online and offline services. Three-quarters of the participants in our study expressed gratitude for this emotional support, which includes telephone greetings, regular home visits, and community activities. Such support is particularly beneficial for those living alone or with a spouse, as it helps alleviate loneliness and foster social inclusion. The continuity of social services further contributes to older adults’ trust and connectedness with the PAT.

Financial benefit is another crucial factor. Existing discussions on smart home technologies have stressed the health-related financial benefits, such as enhanced telehealth services, improved chronic disease management, and reduced healthcare costs [51]. Yet, narratives from participants in this study have highlighted the financial benefits of PAT in their daily lives. PAT continuously connects with and advocates for third-party community and social resources, aiming to provide them greater daily support and better protection of their rights and interests.

Western research raises concerns about a potential divide between technology beneficiaries and those who cannot afford access, thereby exacerbating social inequality [52]. Inclusive service is defined as an egalitarian system that provides clients with fair access to services, fair treatment during services, and an equitable opportunity to quit when desired [53]. The inclusive characteristic is particularly prominent in the context of smart home services in the Chinese mainland, which are viewed as public or quasi-public goods rather than private commodities, reflecting dual development goals of industrial advancement and social welfare [54]. The inclusive services provided by PAT in Jiangmen City effectively address gaps to support older adults.

### 4.3. Contextualizing Smart Home Technology for Older Adults

The differences in smart home technologies between China and the West might be attributed to cultural, economic, and social discrepancies. Chinese Confucian culture emphasizes collectivism and familial responsibility. While PAT supports older adults, it also aids their family members through family-based services. For instance, when older adults require emergency assistance, PAT simultaneously contacts their emergency contacts. In contrast, Western cultures generally prioritize individualism, which results in technologies designed to enhance autonomy and convenience [55]. For example, in one qualitative study examining American older adults’ attitudes toward and perceptions of ‘smart home’ technologies, participants emphasized the importance of technological advancement in providing emergency assistance. However, they also expressed concerns about the technology’s limited human responsiveness and its user-friendliness [56]. Additionally, Western frameworks for smart home applications often emphasize efficiency and technological automation. In contrast, PAT prioritizes service-oriented features, such as emotional support and community engagement, which resonate with Chinese cultural values that emphasize interpersonal relationships and community cohesion. PAT’s emphasis on trust-building through district management systems facilitates connections between service providers and users, in contrast to the more transactional nature of service delivery commonly observed in Western contexts, especially in Western Europe and the United States [55]. Economic conditions further influence these approaches. China’s emphasis on practical, service-oriented features addresses the immediate needs of older adults, while Western countries often invest in advanced technologies for lifestyle enhancement, which requires relatively more financial resources [3]. PAT services prioritize emergency responses to ensure the safety and security of older adults. Although PAT has not yet introduced remote health consultation services, it has made significant strides in related areas, such as health greetings, wellness checks, medication procurement, appointment scheduling, and discharge follow-ups, all designed to support older adults in managing their daily health. This divergence revealed in our study highlights the significance of contextualizing eldercare within cultural and socioeconomic frameworks.

### 4.4. Beyond TAM and UTAUT: Foregrounding Socio-Technical and Cultural Factors

This study highlights the significance of socio-technical and cultural factors in the adoption of smart home technology from the perspectives of Chinese older adults, beyond the two commonly used theories, TAM and UTAUT. These theories emphasize users’ assessments of technology and assume a direct causal influence from intention to actual use behavior, and are criticized for neglecting broader system-level factors that extend beyond the technology and users’ acceptance and satisfaction with it. This research demonstrates that a more comprehensive understanding of individuals’ application or intention to use the technology should also incorporate social-technical systems into its evaluation and use [21]. Technological elements encompass system features and functionality, interoperability, and usability. Organizational aspects and social components include interactions and interpersonal relationships with service providers, social norms, cultural factors, appreciation and trust in the technology, opinions from peers, suggestions from professionals, and so forth. In the Chinese context, TAM and UTAUT cannot account for this because it depends not only on users’ perceptions of the technology’s usefulness or ease of use, but on the socio-cultural embeddedness of service relationships, the district management system, and the alignment of the service model with Confucian values of interdependence. Furthermore, the study necessitates a return to the essence of the humanities to bridge the technology divide, paying more attention to psychological support and social inclusion, and contextualizing smart home technology for older adults, tailored to local economic development and the cultural values and beliefs of communities.

Future PAT services are recommended to better address the multifaceted challenges, including cognitive, technological, psycho-cultural, economic, and ethical dimensions. Addressing the psycho-cultural attitudes that discourage help-seeking behaviors is crucial for improving post-implementation outcomes. Community-based education and empowerment initiatives that encourage a cultural shift towards raising help-seeking awareness, recognizing the significance of formal support, and fostering social inclusion might play a vital role. Multimedia platforms, such as Douyin and WeChat, can be used to further promote the services. At the same time, regarding the significance of social influences, recommendations and promotions from family members, peers, community leaders, and other significant others are vital. For instance, older adult ambassadors can be cultivated to promote services. Organizational support, including person-centered online and offline services and ongoing trust-building practices, is recommended. Furthermore, the agency can establish a regular mechanism for service users to engage in service design and evaluation, thereby improving services to address their needs and increasing their participation.

## 5. Implications

The findings of this study carry several significant implications and can serve as a reference for other countries and regions facing similar challenges in implementing smart home technology. First, an integrated understanding of smart home technology should be cultivated that emphasizes not only the personal and technological dimensions but also the organizational and social dimensions. Organizational support is vital for promoting the adoption of smart home technology among older adults, particularly during the initial implementation stage. Tailored interventions, such as teaching initiatives and cost-effective techniques, are recommended to enhance older adults’ digital skills and self-efficacy, with customized solutions being preferred. Moreover, ongoing trust-building practices are meaningful for minimizing barriers to implementation. Second, enhancing the effectiveness of smart home technology needs more attention. Returning to the essence of the humanities to bridge the technology divide and to make technology better respond to older adults’ needs is key to the design and application of smart home technology, including integrating it with social services, which deserves further exploration. Third, promoting contextual practices is recommended, and diverse social and cultural contexts need to be considered when applying smart home technology for older adults.

## 6. Limitations

First, concerning the methodology, dyadic interviews were applied, in addition to individual interviews, in this study. Dyadic interviews focus on the co-construction of narrative and may result in the potential silencing of one partner’s views, particularly in a cultural context where gender hierarchies within couples may be significant. Second, concerning the reflexivity of the research team, though we strived to mitigate potential bias, the study is inevitably shaped by pre-existing professional frameworks, potentially impacting the way the data were interpreted. The third limitation concerns the relative thinness of some subthemes, such as ethical concerns, which may need further exploration in future research to fully understand their role within the smart home context. Fourth, concerning generalizability, this research is based on PAT services in Jiangmen City, China. Although we have tried to include individuals from diverse demographic backgrounds, the unique cultural, social, and economic context of Jiangmen City may limit the generalizability of the findings and may not reflect the experiences of older adults in other regions of China or different countries. Nevertheless, these findings provide insight into how Chinese older adults perceive and experience smart home technology, thereby shedding light on their needs and strategies for its application, and can serve as a reference for regions and countries with similar socioeconomic and cultural backgrounds.

## 7. Conclusions

Although smart home technology is widely applied in the Chinese eldercare market, its effectiveness from users’ perspectives remains underexplored. This study examines the experiences and perceptions of older adult users in adopting and applying PAT, and presents their perceived biopsychosocial benefits. This study highlights the need to cultivate an integrated understanding of smart home technology, enhance its effectiveness through an integrated service model, and promote cultural and social contextual application among older adults. This knowledge helps inform policymakers, technology designers, and social service providers about the unique needs of older adults and develop strategies for effective support. Future research is recommended to investigate the perspectives and experiences of older adults using smart home technology across different socioeconomic and cultural contexts to gain a deeper understanding of service utilization across countries and regions. A clinical data-mining method is also recommended to investigate the application of smart home technology based on the experiences of older adults.

## Figures and Tables

**Table 1:** Demographic features of the participants.

Participant	Sex	Age	Marital Status	No. of Kids	Occupation Before Retirement	Physical Condition	Pension	Subsidy
***Living alone (n = 11)***								
A1	M	80	Widowed	2	Worker in a real estate company	Diabetes, hypertension	Yes; 4500 ^a^	
A2	M	71	Single	0	Machinist	Normal	No	Poverty subsidy: 1720
A3	F	80	Single	0	Blue-collar worker	Rheumatism	Yes; 3000	
A4	M	67	Divorced	1	Businessman	Glaucoma	No	Poverty subsidy: 1200
A5	F	90	Widowed	3	Worker in a power plant	Normal	Yes; 5000	
A6	F	83	Widowed	4	Worker in a paper factory	Glaucoma, slightly deaf in one ear	Yes; 2450	
A7	F	83	Widowed	5	Peasant	Minor defect in both vision and hearing	No	Government subsidy: 200
A8	F	69	Widowed	3	Blue-collar worker	Normal	Yes; 2700	
A9	F	82	Widowed	3	Machinist	Normal	Yes; 3200	
A10	M	78	Divorced	1	Owner of a metal factory	Minor hearing defects, hypertension, and shoulder periarthritis	Yes; 2200	
A11	M	67	Divorced	1	Technician	Diabetes, vision defect	No	Poverty subsidy: 1430
***Living with a spouse*** ^b^ ***(n = 9)***								
B1_M	M	84	Married	1	Driving coach	Walking independently but at a slow pace	Yes; 3500	
B1_F	F	79	Married	/	Bus ticket seller	Normal	Yes; 3000	
B2_M	M	84	Married	3	Blue-collar worker	Walking independently but at a slow pace, with a minor vision defect	Yes; 4290	
B2_F	F	80	Married	/	/	Normal	No	
B3_M ^c^	M	85	Married	1	Association chairman	Foot pain, knee swelling, and partial blindness in the right eye	Yes; 3200	
B4_M	M	80	Married	1	Construction Manager	Normal	Yes; 1800	
B4_F	F	80	Married	/	Peasant	Knee pain, diabetes	No	Government subsidy: 203
B5_M	M	79	Married	2	Bus driver	Suspected of having mild dementia	Yes; 3600	
B5_F	F	75	Married	/	Blue-collar worker	Normal	Yes; 3000	
***Living with family members (n = 8)***								
C1	F	87	Widowed	2	Worker in an electronics factory	Minor hearing defect, rheumatism	Yes; 3200	
C2	F	90	Widowed	3	/	Minor hearing defect, lower back pain, foot pain, gout, and bone hyperplasia	/	
C3	M	83	Married	1	Blue-collar worker	Normal	Yes; 3000	
C4	M	60	Married	1	Small shop owner	Needing a wheelchair for mobility	No	Disability allowance: 1000
C5	F	82	Widowed	2	Salesperson	Degenerative arthritis, hypertension, and diabetes	Yes; 3600	
C6	M	79	Widowed	4	Bus driver	Gout and hypertension	Yes; 3500	
C7	M	86	Widowed	4	Mason	Partial blindness, reduced hearing	Yes; 1500	
C8	F	63	Married	1	Blue-collar worker	Muscle weakness	No	Disability allowance: 480

^a^. The currency of pensions and subsidies is Chinese Yuan (CNY). ^b^. ‘Living with a spouse’ means that the family consists of only two older adults living together. ^c^. B3_M’s wife was not at home during our home visit and was thus excluded from our participant list.

**Table 2:** Details of the overarching themes and subthemes.

Overarching Themes	Subthemes
Multifaceted challenges of applying PAT	Cognitive insufficiency Technological barriers Psycho-cultural resistance Economic concerns Ethical concerns
Bridging the technology divide	Empowering the use of PAT Improving effectiveness through the integration of technology and social services Enhancing accessibility through inclusive services

## Data Availability

The data presented in this study are available on request from the corresponding author due to data privacy and confidentiality.

## References

[1] Chen H., Hagedorn A., An N. (2023). The development of smart eldercare in China. Lancet Reg. Health West. Pac..

[2] Yu Y. (2024). ‘Smartness’ narratives: A critical discourse analysis of smart eldercare in urban China. Trans. Inst. Br. Geogr..

[3] Marikyan D., Papagiannidis S., Alamanos E. (2019). A systematic review of the smart home literature: A user perspective. Technol. Forecast. Soc. Change.

[4] Vrančić A., Zadravec H., Orehovački T. (2024). The Role of Smart Homes in Providing Care for Older Adults: A Systematic Literature Review from 2010 to 2023. Smart Cities.

[5] Islam S.M.S., Halooq A., Dening J., Uddin R., Laranjo L., Chow C.K., Maddison R. (2022). Healthcare providers’ perspectives on using smart home systems to improve self-management and care in people with heart failure: A qualitative study. Int. J. Med. Inform..

[6] Lolich L., Riccò I., Deusdad B., Timonen V. (2019). Embracing technology? Health and Social Care professionals’ attitudes to the deployment of e-Health initiatives in elder care services in Catalonia and Ireland. Technol. Forecast. Soc. Change.

[7] Facchinetti G., Petrucci G., Albanesi B., De Marinis M.G., Piredda M. (2023). Can smart home technologies help older adults manage their chronic condition? A systematic literature review. Int. J. Environ. Res. Public Health.

[8] Mois G., Fortuna K.L. (2020). Visioning the Future of Gerontological Digital Social Work. J. Gerontol. Soc. Work.

[9] Ghafurian M., Wang K., Dhode I., Kapoor M., Morita P.P., Dautenhahn K. (2023). Smart home devices for supporting older adults: A systematic review. IEEE Access.

[10] Park E., Kim S., Kim Y., Kwon S.J. (2018). Smart home services as the next mainstream of the ICT industry: Determinants of the adoption of smart home services. Univers. Access Inf. Soc..

[11] Tian Y.J., Felber N.A., Pageau F., Schwab D.R., Wangmo T. (2024). Benefits and barriers associated with the use of smart home health technologies in the care of older persons: A systematic review. BMC Geriatr..

[12] Lee L.N., Kim M.J. (2020). A Critical Review of Smart Residential Environments for Older Adults with a Focus on Pleasurable Experience. Front. Psychol..

[13] Tsertsidis A., Kolkowska E., Hedström K. (2019). Factors influencing seniors’ acceptance of technology for ageing in place in the post-implementation stage: A literature review. Int. J. Med. Inform..

[14] Felber N.A., Tian Y.J., Pageau F., Elger B.S., Wangmo T. (2023). Mapping ethical issues in the use of smart home health technologies to care for older persons: A systematic review. BMC Med. Ethics.

[15] Sundgren S., Stolt M., Suhonen R. (2020). Ethical issues related to the use of gerontechnology in older people care: A scoping review. Nurs. Ethics.

[16] Davis F.D. (1989). Perceived usefulness, perceived ease of use, and user acceptance of information technology. MIS Q..

[17] Venkatesh V., Morris M.G., Davis G.B., Davis F.D. (2003). User acceptance of information technology: Toward a unified view. MIS Q..

[18] Venkatesh V., Thong J., Xu X. (2016). Unified Theory of Acceptance and Use of Technology: A Synthesis and the Road Ahead. J. Assoc. Inf. Syst..

[19] Momani A.M. (2020). The Unified Theory of Acceptance and Use of Technology: A New Approach in Technology Acceptance. Int. J. Sociotechnol. Knowl. Dev..

[20] Fishbein M., Ajzen I. (2010). Predicting and Changing Behavior: The Reasoned Action Approach.

[21] Lee A.T., Ramasamy R.K., Subbarao A. (2025). Understanding psychosocial barriers to healthcare technology adoption: A review of TAM technology acceptance model and unified theory of acceptance and use of technology and UTAUT frameworks. Healthcare.

[22] Shachak A., Kuziemsky C., Petersen C. (2019). Beyond TAM and UTAUT: Future directions for HIT implementation research. J. Biomed. Inform..

[23] Schulz R., Wahl H.-W., Matthews J.T., Dabbs A.D.V., Beach S.R., Czaja S.J. (2015). Advancing the aging and technology agenda in gerontology. Gerontologist.

[24] Schroeder T., Dodds L., Georgiou A., Gewald H., Siette J. (2023). Older adults and new technology: Mapping review of the factors associated with older adults’ intention to adopt digital technologies. JMIR Aging.

[25] Ekaimi S., Utomo P., Gunawan D., Jimmy S.Y., Christian M. (2024). Examining the factors influencing teleconsultation adoption during the pandemic using the tam model. Glob. Bus. Financ. Rev. (GBFR).

[26] Jamwal R., Jarman H.K., Roseingrave E., Douglas J., Winkler D. (2022). Smart home and communication technology for people with disability: A scoping review. Disabil. Rehabil. Assist. Technol..

[27] Kulurkar P., Dixit C.K., Bharathi V., Monikavishnuvarthini A., Dhakne A., Preethi P. (2023). AI based elderly fall prediction system using wearable sensors: A smart home-care technology with IOT. Meas. Sens..

[28] Zhu H., Walker A. (2018). The gap in social care provision for older people in China. Asian Soc. Work. Policy Rev..

[29] Turjamaa R., Pehkonen A., Kangasniemi M. (2019). How smart homes are used to support older people: An integrative review. Int. J. Older People Nurs..

[30] Ghorayeb A., Comber R., Gooberman-Hill R. (2021). Older adults’ perspectives of smart home technology: Are we developing the technology that older people want?. Int. J. Hum.-Comput. Stud..

[31] Denzin N.K., Lincoln Y.S. (2024). The SAGE Handbook of Qualitative Research.

[32] Saunders B., Sim J., Kingstone T., Baker S., Waterfield J., Bartlam B., Burroughs H., Jinks C. (2018). Saturation in qualitative research: Exploring its conceptualization and operationalization. Qual. Quant..

[33] Clarke V., Braun V. (2017). Thematic Analysis. J. Posit. Psychol..

[34] Lincoln Y.S., Lynham S.A., Guba E.G. (2018). Paradigmatic controversies, contradictions, and emerging confluences, revisited. The Sage Handbook of Qualitative Research.

[35] Philip N.Y., Rodrigues J.J.P.C., Wang H., Fong S.J., Chen J. (2012). Internet of Things for In-Home Health Monitoring Systems: Current Advances, Challenges and Future Directions. IEEE J. Sel. Areas Commun..

[36] Moraitou M., Pateli A., Fotiou S. Smart health caring home: A systematic review of smart home care for elders and chronic disease patients. Proceedings of the GeNeDis 2016: Geriatrics.

[37] Naseer F., Addas A., Tahir M., Khan M.N., Sattar N. (2025). Integrating generative adversarial networks with IoT for adaptive AI-powered personalized elderly care in smart homes. Front. Artif. Intell..

[38] Vadisetty R., Polamarasetti A. (2025). Regulatory Framework for Digital Health, Data Privacy, and Cybersecurity. Sustainable Healthcare Systems in Africa.

[39] Mani Z., Chouk I. (2017). Drivers of consumers’ resistance to smart products. J. Mark. Manag..

[40] Wang S. (2001). The help-seeking relationship in Chinese society. Sociol. Stud..

[41] Fei X., Hamilton G.G., Zheng W. (1992). From the Soil: The Foundations of Chinese Society.

[42] Tian B., Zhong Z. (2009). The ideology of social welfare socialization: Four-dimensional analysis frame of welfare pluralism. Explor. Free Views.

[43] Wen Z. (2017). Government Purchase of Services in China: Similar Intentions, Different Policy Designs. Public Adm. Dev..

[44] Chan C.K., Lei J. (2017). Contracting social services in China: The case of the Integrated Family Services Centres in Guangzhou. Int. Soc. Work.

[45] Kong L., Woods O. (2018). Smart eldercare in Singapore: Negotiating agency and apathy at the margins. J. Aging Stud..

[46] Moor N., Mohammadi M., Biloria N. (2020). Grey Smart Societies: Supporting the Social Inclusion of Older Adults by Smart Spatial Design. Data-Driven Multivalence in the Built Environment.

[47] Cao L., Feng S., Chen K., Wu X., Jia Y. (2025). An ethical model for smart home-based elder care. Nurs. Ethics.

[48] Aw S., Koh G.C.H., Tan C.S., Wong M.L., Vrijhoef H.J.M., Harding S.C., Geronimo M.A.B., Hildon Z.J.L. (2019). Theory and Design of the Community for successful ageing (ComSA) program in Singapore: Connecting BioPsychoSocial health and quality of life experiences of older adults. BMC Geriatr..

[49] Luo J., Guo Y., Tian Z. (2024). Loneliness or Sociability: The Impact of Social Participation on the Mental Health of the Elderly Living Alone. Health Soc. Care Community.

[50] Vogelsang E., Moorman S. (2024). High Impact Activities: Assessing the Association Between Social Participation Frequency and Cognition. Innov. Aging.

[51] Rattanawiboomsom V., Talpur S.R. (2023). Enhancing Health Monitoring and Active Aging in the Elderly Population: A Study on Wearable Technology and Technology-Assisted Care. Int. J. Online Biomed. Eng..

[52] Dermody G., Fritz R., Glass C., Dunham M., Whitehead L. (2021). Factors influencing community-dwelling older adults’ readiness to adopt smart home technology: A qualitative exploratory study. J. Adv. Nurs..

[53] Fisk R.P., Dean A.M., Alkire L., Joubert A., Previte J., Robertson N., Rosenbaum M.S. (2018). Design for service inclusion: Creating inclusive service systems by 2050. J. Serv. Manag..

[54] Zhang Q., Li M., Wu Y. (2020). Smart home for elderly care: Development and challenges in China. BMC Geriatr..

[55] Del Rio D.D.F., Sovacool B.K., Griffiths S. (2021). Culture, energy and climate sustainability, and smart home technologies: A mixed methods comparison of four countries. Energy Clim. Change.

[56] Choi Y.K., Thompson H.J., Demiris G. (2021). Internet-of-Things Smart Home Technology to Support Aging-in-Place: Older Adults’ Perceptions and Attitudes. J. Gerontol. Nurs..

